# Predictive factors for massive hemorrhage in women with retained products of conception: a prospective study

**DOI:** 10.1038/s41598-022-15564-1

**Published:** 2022-07-13

**Authors:** Reina Sonehara, Tomoko Nakamura, Akira Iwase, Kazuki Nishida, Sachiko Takikawa, Mayuko Murakami, Sayako Yoshita, Ayako Muraoka, Natsuki Miyake, Natsuki Nakanishi, Satoko Osuka, Maki Goto, Hiroaki Kajiyama

**Affiliations:** 1 Department of Obstetrics and Gynecology, Nagoya University Graduate School of Medicine, 65 Tsurumai-cho, Showa-ku, Nagoya, 466-8550 Japan; 2 Department of Obstetrics and Gynecology, Gunma University Graduate School of Medicine, 3-39-22, Showa-machi, Maebashi, 371-8511 Japan; 3 Department of Biostatistics Section, Center for Advanced Medicine and Clinical Research, Nagoya University Hospital, 65 Tsurumai-cho, Showa-ku, Nagoya, 466-8550 Japan; 4 Clinic Mama, 3-34-1, Imajuku, Ogaki, Gifu 503-0807 Japan; 5 Department of Maternal and Perinatal Medicine, Nagoya University Hospital, 65 Tsurumai-cho, Showa-ku, Nagoya, 466-8550 Japan

**Keywords:** Diseases, Risk factors

## Abstract

Retained products of conception (RPOC) is a common cause of postpartum bleeding, which may be life-threatening; however, no evidence-based guidelines exist to assist in evaluating the risk of massive hemorrhage in women with RPOC. In this prospective study, we aimed to evaluate the predictive factors for massive hemorrhage in women with RPOC. The primary and secondary endpoints were to validate the usefulness of power Doppler color scoring (PDCS) in evaluating hypervascularity and to identify other predictive factors (such as maximum RPOC diameter and serum βhCG and Hb level at first visit), respectively. Among the 51 women with RPOC included in this study, 16 (31.5%) experienced massive hemorrhage during follow-up. None of the women with PDCS 1 or 2 (18) experienced massive hemorrhage, whereas 16 (48.5%) women with PDCS 3 or 4 (33) did. Multiple logistic regression analysis showed that the odds ratio [95% confidence interval] (*P* value) for PDCS, assisted reproductive technology (ART), and low serum hemoglobin (Hb) levels were 22.39 [2.25 − 3087.92] (*P* = 0.004), 5.72 [1.28 − 33.29] (*P* = 0.022), and 4.24 [0.97 − 22.99] (*P* = 0.056), respectively. Further, the decision tree method identified PDCS, ART, and low serum Hb levels as potential predictive factors for massive hemorrhage. This study identified PDCS as useful predictor of massive hemorrhage in women with RPOC. With additional inclusion of factors such as ART and low serum Hb levels, the risk of massive hemorrhage may be effectively evaluated, leading to better management of women of reproductive age.

## Introduction

Retained products of conception (RPOC) refers to intrauterine tissues of placental origin^1^ that persist after delivery or spontaneous/induced abortion^2–4^; RPOC is one of the most common causes of postpartum bleeding, which may be life-threatening^5,6^. The management of patients with RPOC is uncertain due to lack of evidence-based guidelines (to assess the risk of massive hemorrhage) and optimal treatment protocols^7^.

Profuse hemorrhage may require resuscitation with blood transfusions, interventional radiology procedures, surgical resection, or hysterectomy to control bleeding^8–10^. In general, uterine artery embolization (UAE) and hysteroscopic resection (HR) are considered safe and efficient interventional strategies for managing massive hemorrhage^7,11–13^. Expectant management may also be achieved^14,15^; however, it is difficult to predict the probability of massive hemorrhage during the follow-up on the first visit of the patient with RPOC.

Hypervascularity is a characteristic feature of RPOC. Enhanced vascularity is also observed in uterine arterio-venous malformation (UAVM), frequently causing a diagnostic challenge. However, some report that RPOC can be differentiated from UAVMs by sonography by detecting the vascular endometrial component in RPOC, whereas the myometrium is mainly involved in UAVMs^1^. Three-dimensional computed tomography is also reported to be effective in distinguishing RPOC from UAVM^16^. The degree of vascularity in RPOC compared with that in the adjacent myometrium can range from avascular to markedly hypervascular^5^. Power Doppler color scoring (PDCS) is a four-level grading system used to evaluate vascularity^17^. A PDCS score ranged from 1 to 4, where 1 was given when no blood was found within the RPOC, and 4 was given when a very strong blood flow was detected. PDCS has been reported to predict the likelihood of successful expectant management after incomplete miscarriage^18^. In a retrospective case–control study, PDCS was also suggested to be useful in predicting the need for surgical intervention in women with RPOC after delivery or spontaneous/induced abortion^19^. Another retrospective study identified the ultrasound vascularity scores, serum hemoglobin (Hb) levels, and endometrial thickness as significant predictors for surgical intervention in women with RPOC^20^.

Since only few prospective studies have been conducted, in this study, we prospectively evaluated the predictive factors for massive hemorrhage in women with RPOC. This study also aimed to validate the potential of PDCS as a predictive model for massive hemorrhage.

## Methods

### Patients

We enrolled women diagnosed with RPOC at Nagoya University Hospital, Nagoya, Japan, between February 2017 and May 2020. The study was conducted in accordance with the ethical principles of the Declaration of Helsinki and approved by the Institutional Review Board of Nagoya University Hospital (#2016-0448). All patients included in this study provided written informed consent.

RPOC was diagnosed using transvaginal ultrasonography as persistent intrauterine tissue after spontaneous or induced abortion, or delivery. The diagnosis was confirmed with other diagnostic imaging techniques, such as three-dimensional computed tomography, when equivocal. Massive hemorrhage was defined as the presence of active bleeding and at least one of the following: loss of consciousness, shock index greater than 1, or serum hemoglobin (Hb) level < 10 g/dL. Women included in this study were recorded of massive hemorrhage which occurred after their first visit until the RPOC resolution. Women who presented with massive hemorrhage upon their first visit to our hospital were excluded, as well as women with a history of intrauterine diseases (endometrial polyps and submucous myomas).

### Endpoints

The primary endpoint was to validate the usefulness of PDCS as a predictive factor for massive hemorrhage in women with RPOC. The secondary endpoint was the identification and evaluation of other potential predictive factors.

### Protocol

Women with RPOC underwent expectant management unless they experienced massive hemorrhage. Expectant management is defined as a careful “watch” approach. Massive hemorrhage was treated using UAE and/or HR. UAE prior to HR was performed because HR would be limited by massive bleeding, which creates poor visual field and increases the risk of bleeding from the hypervascular uterus. HR was also performed for non-bleeding, but persistent residual tumors measuring ≥ 2 cm. Complete resolution was defined as no evidence of RPOC on transvaginal ultrasound and complete regression (< 5 IU/mL) of serum β-human chorionic gonadotropin (βhCG). At the first visit, women were interviewed for their past pregnancy history and details of the preceding pregnancy responsible for RPOC. The latter included the method for achieving pregnancy, outcome of the pregnancy, trimester at delivery or abortion, and whether intrauterine interventions, such as dilatation and curettage and/or manual removal of the placenta, were required. The clinical findings at the first visit were also obtained. PDCS and maximum RPOC diameter by transvaginal ultrasound, serum βhCG level, and Hb level were recorded. The length of follow-up was calculated as the time from first visit to RPOC resolution. The time from delivery to massive hemorrhage, complete regression (< 5 IU/mL) of serum βhCG level, and RPOC resolution were also calculated.

### Sonography

RPOC was diagnosed based on the presence of a measurable focus of hyperechoic material within the endometrial cavity on two-dimensional gray-scale transvaginal ultrasonography. A subjective qualitative assessment of flow within the RPOC was performed. The transvaginal ultrasound settings were adjusted to allow maximal sensitivity to blood flow. The ultrasonic frequency was set to 8.0 MHz, and the power Doppler gain was reduced until the artifacts disappeared.

The use of subjective semi-quantitative assessment of flow to describe vascular features has been suggested in previous reports^17,18,20,21^. In this study, PDCS was used to describe the amount of blood flow to the residual tissue. PDCS ranges from a score of 1 to 4, with a score of 1, 2, 3, and 4 indicating no detectable blood flow, minimal flow, moderate flow, and highly vascular flow, respectively^17,18,21^. The PDCS refers only to the color Doppler image and not the Doppler shift spectrum.

### Statistical analysis

Data were compared between women who did not experience massive hemorrhage and those who did (no hemorrhage group vs. hemorrhage group). Continuous variables are presented as medians with minimum and maximum ranges and analyzed using the Mann–Whitney U test. Categorical variables are presented as frequencies with proportions and analyzed using the Fisher’s exact test. For a preliminary analysis to determine the optimal cutoff for each predictive factor for massive hemorrhage, receiver operating characteristic (ROC) curves and area under the curve (AUC) were calculated. For the main analysis, we performed logistic regression analysis to estimate the odds ratio (OR) with a 95% confidence interval (CI) of the predictive factors for massive hemorrhage. Because of the small number of cases, the logistic regression model was corrected using Firth’s method, and four models were created for multivariate analysis. Furthermore, a decision tree analysis was performed to assess predictive factors from multiple perspectives. In all analyses, a *P* value < 0.05 was considered statistically significant. Microsoft Excel, IBM SPSS Statistics for Windows, version 26.0 (IBM Corp., Armonk, NY, USA) and R 3.6.1 for Mac were used to generate graphs and perform statistical analyses.

## Results

**Figure 1:**
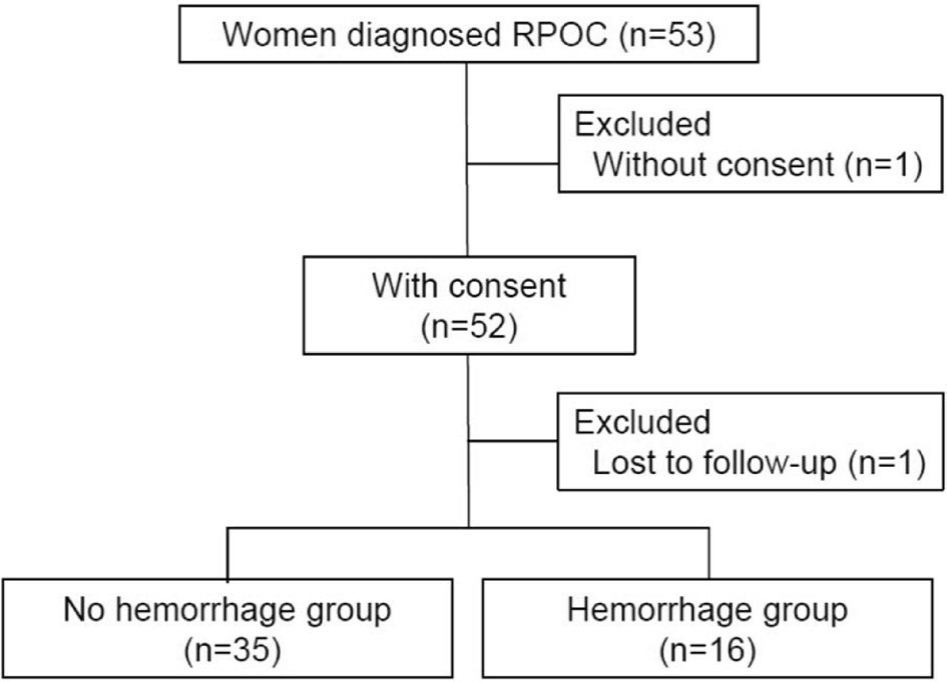
Patient flow chart. A total of 53 women diagnosed with RPOC were recruited. Moreover, 68.6% (35/51) of the women did not experience massive hemorrhage (no hemorrhage group), whereas 31.3% (16/51) required intervention for massive hemorrhage (hemorrhage group).

A total of 53 women with RPOC were recruited in this study; one patient refused to consent, whereas another was diagnosed with uterine disease during follow-up and underwent surgery. Among the 51 patients, 35 (68.6%) completed expectant management with no massive hemorrhage (no hemorrhage group), whereas 16 (31.3%) developed massive hemorrhage, requiring interventional treatments (hemorrhage group) (Fig. 1).

### Patient characteristics

Table 1 shows the comparison of the clinical characteristics of women with RPOC between the hemorrhage and no hemorrhage groups. Significant differences were observed in age (*P* = 0.033), method of achieving pregnancy (*P* = 0.003), and findings (maximum RPOC diameter [*P* = 0.03], PDCS [*P* = 0.005], serum βhCG levels [*P* = 0.007], and serum Hb levels [*P* = 0.005]) at the first visit. Notably, no women with a PDCS of 1 or 2 (18) experienced massive hemorrhage, whereas 48.5% (16/33) of women with a PDCS of 3 or 4 did. The proportion of assisted reproductive technology (ART) was significantly higher in the hemorrhage group than in the no hemorrhage group (81.3% [13/16] vs. 34.3% [12/35], *P* = 0.003). Serum Hb levels were lower in the hemorrhage group (9.6 g/dL [6.2–13.9]) than in the no hemorrhage group (11.7 g/dL [8.3–14.7]). The trimester of delivery or abortion of the preceding pregnancy responsible for RPOC was not significantly different between the two groups (*P* = 0.37). Furthermore, we found no significant differences in the length of follow-up and the time to complete regression of serum βhCG levels between the two groups (*P* = 0.29 and *P* = 0.52).

**Table 1:** Patient characteristics. Comparison between the no hemorrhage and hemorrhage groups.

	No hemorrhage group (n = 35)	Hemorrhage group (n = 16)	*P* value
Age (y)*	32 (22–42)	36.5 (26–48)	0.033^†^
**Past pregnancy (%)**			
Yes	18 (51)	8 (50)	1.0**
No	17 (49)	8 (50)	
**Preceding pregnancy (%)**^‡^			
Method			
Ni	23 (66)	3 (19)	0.003**
ART	12 (34)	13 (81)	
IVF-ET	11	13	
IUI	1	0	
Outcome			
SA	20 (57)	5 (31)	0.19**
AA	5 (14)	4 (25)	
VD	10 (29)	6 (36)	
CS	0 (0)	1 (8)	
Trimester at delivery			
First	20 (57)	6 (38)	0.37**
Second	6 (17)	3 (19)	
Third	9 (26)	7 (43)	
Intrauterine intervention^§^			
Yes	17 (49)	7 (43)	0.77**
No	18 (51)	9 (57)	
**Findings at first visit**			
Maximum diameter (mm)*	28 (6.9–68)	40 (17–71)	0.03^†^
PDCS (%)			
1	10 (29)	0 (0)	0.005**
2	8 (22)	0 (0)	
3	7 (20)	7 (44)	
4	10 (29)	9 (56)	
βhCG (IU/L)*	9.2 (1.2–3,244.8)	50.6 (2.5–5,339.7)	0.007^†^
Hb (g/dL)*	11.7 (8.3–14.7)	9.6 (6.2–13.9)	0.005^†^
**Clinical course**			
HR (%)	1 (3)	13 (81)	–
Length of follow-up (days)*			
Time from delivery (days)	40 (10–340)	34 (17–202)	0.77^†^
To massive hemorrhage*	–	32.5 (6–74)	–
To βHCG regression*	58 (26–146)	64.5 (16–91)	0.52^†^
To RPOC resolution*	103 (53–405)	75 (31–258)	0.29^†^

Ni, natural intercourse; ART, assisted reproductive technology; IVF-ET, in vitro fertilization-embryo transfer; IUI, intrauterine insemination; SA, spontaneous abortion; AA, artificial abortion; VD, vaginal delivery; CS, cesarean section; PDCS, power Doppler color scoring; βhCG, serum β-human chorionic gonadotropin; Hb, serum hemoglobin; HR, hysteroscopic resection.

*median (range), ^‡^pregnancy responsible for RPOC, ^§^dilatation and curettage and manual removal of placenta, ^†^Mann–Whitney *U* Test, **Fisher exact test. Statistical significance was set at a *P* value of < 0.05.

### Predictive factors for massive hemorrhage

Using ROC curve analysis, the serum Hb and βhCG levels and maximum RPOC diameter at first visit were assessed as predictive factors for massive hemorrhage (Supplementary Fig. S1). Low serum Hb levels (AUC, 0.75; sensitivity, 75.0%; specificity, 70.6%; *P* = 0.003) and high βhCG levels (AUC, 0.73; sensitivity, 68.8%; specificity, 67.6%; *P* = 0.001) were identified to be highly significant predictive factors for massive hemorrhage. A larger maximum RPOC diameter (AUC, 0.68; sensitivity, 56.3%; specificity, 44.1%; *P* = 0.019) was also a significant predictor of massive hemorrhage. The optimal cutoff values for serum Hb levels, βhCG levels, and maximum RPOC diameter were 10.8 g/dl, 25.1 IU/L, and 30.5 mm, respectively (Supplementary Table S2).

Univariate logistic regression analysis showed significant differences in PDCS (*P* = 0.000), ART (*P* = 0.002), serum Hb levels (*P* = 0.005), and serum βhCG levels (*P* = 0.014) as predictive factors for massive hemorrhage; however, there were no significant differences in diameter (*P* = 0.38) and pregnancy trimester (*P* = 0.16) (Table 2). Multivariable analysis included four models and PDCS and ART were included as explanatory variables in all models. The third explanatory variable was selected from Hb, βhCG, diameter, and pregnancy trimester. In the main model, the OR with 95% CI (*P* value) for PDCS, ART, and serum Hb levels were 22.39 [2.25 − 3,087.92] (*P* = 0.004), 5.72 [1.28 − 33.29] (*P* = 0.022), and 4.24 [0.97 − 22.99] (*P* = 0.056), respectively. PDCS consistently showed significant differences in all models (main model, *P* = 0.004; model 1, *P* = 0.004; model 2, *P* = 0.002; model 3, *P* = 0.002), whereas ART showed significant differences in three models (main model, *P* = 0.022; model 2, *P* = 0.031; model 3, *P* = 0.01).

**Table 2:** Odds ratio of variables for massive hemorrhage in univariable and multivariable logistic regression models. Multivariable analysis was evaluated in four models.

Variable	OR [95% CI] (*P* value) in univariate analysis	Adjusted OR [95% CI] (*P* value) in multivariable analysis^†^			
		Main model	Model 1	Model 2	Model 3
PDCS	34.89 [4.13–4,573.57] (0.000)	22.39 [2.25–3,087.92] (0.004)	20.43[2.25–2,710.32] (0.004)	29.95 [2.79–4,171.13] (0.002)	22.40 [2.49–2,966.14] (0.002)
ART	7.25 [2.02–32.59] (0.002)	5.72 [1.28–33.29] (0.022)	4.17 [0.94–21.69] (0.06)	4.83 [1.15–24.34] (0.031)	7.40 [1.56–51.58] (0.01)
Hb	5.68 [1.66–22.61] (0.005)	4.24 [0.97–22.99] (0.056)	–	–	–
βhCG	1.68 [0.53–5.51] (0.014)	–	1.86 [0.41–8.35] (0.414)	–	–
Diameter	4.45 [1.35–16.36] (0.38)	–	–	0.76 [0.17–3.47] (0.715)	–
Trimester	1.58 [0.83–3.09] (0.16)	–	–	–	2.35 [0.98–6.98] (0.057)

OR, odds ratio; CI, confidence interval; PDCS, power Doppler color scoring; ART, assisted reproductive technology; Hb, serum hemoglobin; βhCG, serum β-human chorionic gonadotropin. ^†^Logistic regression model with Firth’s correction method. *P* value of < 0.05 were considered statistically significant.

**Figure 2:**
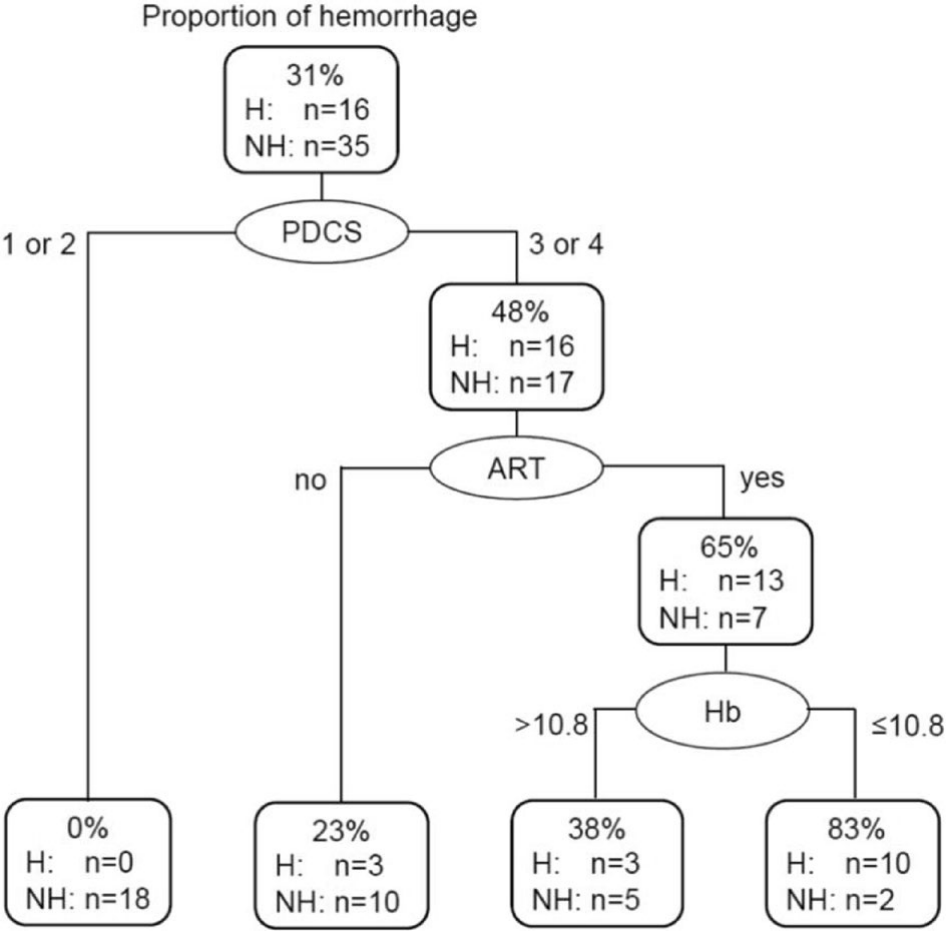
Decision tree model of predictive factors for massive hemorrhage. Decision tree method identified PDCS, ART, and serum Hb levels as effective variables in massive hemorrhage prediction. H: hemorrhage, NH: no hemorrhage, PDCS: power Doppler color scoring, ART: assisted reproductive technology, Hb: serum hemoglobin. βhCG: serum β-human chorionic gonadotropin, ROC: receiver operating characteristic.

The decision tree method identified PDCS, ART, and serum Hb levels as potential predictive factors for massive hemorrhage (Fig. 2). Eighty-three percent of women with RPOC were suggested to have massive hemorrhage if they presented with all three factors of PDCS 3 or 4, ART, and low serum Hb levels.

## Discussion

In the present study, PDCS was identified as the most prominent predictive factor for massive hemorrhage in women with RPOC. Our results support expectant management for women with a PDCS of 1 or 2 because the risk of massive hemorrhage is low, whereas those with a PDCS score of 3 or 4 should be carefully considered for immediate surgical intervention. This is consistent with the findings of a previous report that suggested expectant management and surgical intervention for patients with RPOC of PDCS 1 or 2 and 3 or 4, respectively^19^. Although HR and UAE are commonly reported as efficient strategies for RPOC without detrimental effects on fertility, we found that approximately half of the women with a PDCS of 3 or 4 did not experience massive hemorrhage requiring intervention. The presence of other predictive factors for massive bleeding in addition to PDCS would be able to better screen women with RPOC who would require surgical intervention, thereby leading to a more timely intervention.

This study suggests that ART may also be a potential predictive factor for massive hemorrhage. To the best of our knowledge, this is the first report to describe the association between ART and the risk of massive hemorrhage in women with RPOC. ART has been associated with placenta-mediated complications. Esh-Broder et al. reported that women who conceived with ART had a 13-fold increased risk of placenta accreta spectrum compared to women who conceived naturally^22^. Tourette et al. demonstrated an increased number of in vitro fertilization (IVF) cases in women with placenta accreta without prior cesarean delivery^23^. Although not specifically with RPOC, ART has also been associated with general postpartum hemorrhage^24,25^. The IVF protocol has been suggested to change the endometrial environment, or to interfere with the formation of maternal–fetal interface in early stage implantation^22,25^. ART may play a role in the development of RPOC through similar mechanisms, however, further studies are required.

We have also identified low serum Hb levels at first visit as a potential predictive factor for massive hemorrhage after PDCS and ART. The amount of bleeding at abortion or delivery may correlate better with the risk for massive hemorrhage. However, as a university hospital, most of our patients are referred from other institutions, lacking accurate information on the amount of bleeding. Therefore, we were unable to analyze the effect of the amount of bleeding. Additionally, we speculated that trimester at delivery or abortion, serum βhCG levels, and maximum RPOC diameter may be clinically important factors. A previous report suggested that the disappearance of placental blood flow in the conservative management of placenta accreta was almost coincident with a decrease in serum βhCG level^26^. Matsumura et al. reported that ultrasonographic assessment of RPOC focusing on vascularity in combination with size was essential for detecting the high risk of severe postpartum hemorrhage (SPPH) in women with RPOC^27^. Kobayashi et al. also suggested that the long axis of RPOC is a simple indicator for predicting women with SPPH who would require invasive procedures^28^. However, in our analysis, consistent significant differences in these factors were not observed between the two groups of patients. We also evaluated the RPOC diameter as a predictive factor of massive hemorrhage separately for abortion or term delivery. We found no significant differences between the hemorrhage and no hemorrhage group of women after abortion. Although we noted a trend for greater RPOC diameter in the hemorrhage group compared to the no hemorrhage group of women after term delivery, our results failed to show a significance. The prediction utility of these factors may be better judged in further studies involving a large patient population.

This study has few limitations. Although this was a prospective study, it was a single-center study and the number of cases was small. A larger study may identify other factors allowing more accurate prediction for massive hemorrhage. A scoring system would also be useful for better patient management in a clinical setting. A multicenter study consisting of more cases may enable the development of such scoring system for assessing the risk of massive hemorrhage due to RPOC.

## Conclusion

This study demonstrated that PDCS is the most useful predictive factor for massive hemorrhage in women with RPOC. By considering other predictive factors, such as ART and low serum Hb levels, cases with a higher risk of massive hemorrhage may be effectively detected and better managed.

## Supplementary Information

Supplementary Information.

## Data Availability

The datasets generated and/or analyzed during the current study are available from the corresponding author upon reasonable request.
