# Supplementary material for: Predictive factors for massive hemorrhage in women with retained products of conception: a prospective study
Source: Sci Rep. 2022 Jul 13;12:11859. doi: 10.1038/s41598-022-15564-1 (PMC9279309; doi:10.1038/s41598-022-15564-1)
Supplement: Supplementary file 1 — Supplementary Information. [file 41598_2022_15564_MOESM1_ESM.pdf]

Supplementary Figure S1.
ROC curve in the assessment of prediction factors for massive hemorrhage.

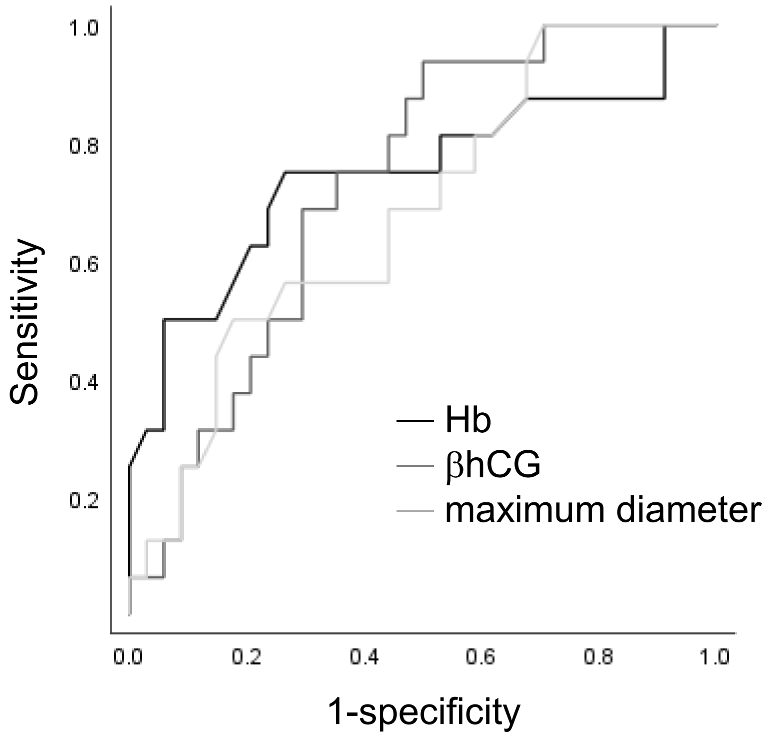


ROC curve were calculated to determine the optimal cutoff for each predictive factors of massive hemorrhage such as serum Hb level, serum βhCG level, and maximum diameter of RPOC. Hb: serum hemoglobin, βhCG: serum beta-human chorionic gonadotropin.

Supplementary Table S2. Cut off values obtained by ROC analysis.

| | AUC (95% CI) | P value | Cut off value | Sensitivity (%) | Specificity (%) |
|---|---|---|---|---|---|
| Hb (g/dL) | 0.75 (0.58-0.91) | 0.003 | 10.8 | 75.0 | 70.6 |
| βhCG (IU/L) | 0.73 (0.59-0.87) | 0.001 | 25.1 | 68.8 | 67.6 |
| Maximum diameter (mm) | 0.68 (0.53-0.84) | 0.019 | 30.5 | 56.3 | 44.1 |

Low serum Hb levels (<10.8) may predict massive hemorrhage (AUC, 0.75; sensitivity, 75.0%; specificity, 70.6%; *P*=0.003). High βhCG (>25.1) and large diameter (>30.5) may also predict massive hemorrhage (βhCG: AUC, 0.73; sensitivity, 68.8%; specificity, 67.6%; *P*=0.001, Diameter: AUC, 0.68; sensitivity, 56.3%; specificity, 44.1%, p=0.019). Hb: serum hemoglobin, βhCG: serum beta-human chorionic gonadotropin, AUC: area under the curve. *P* value <0.05 were considered statistically significant.
